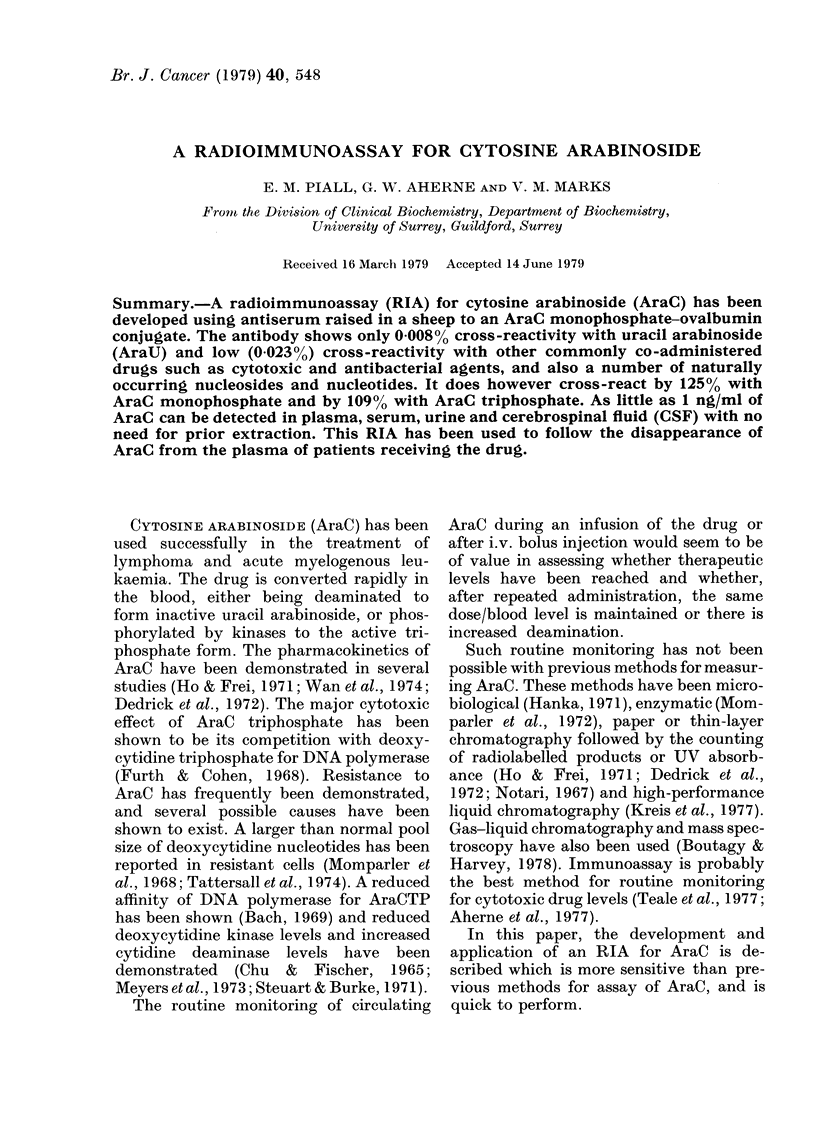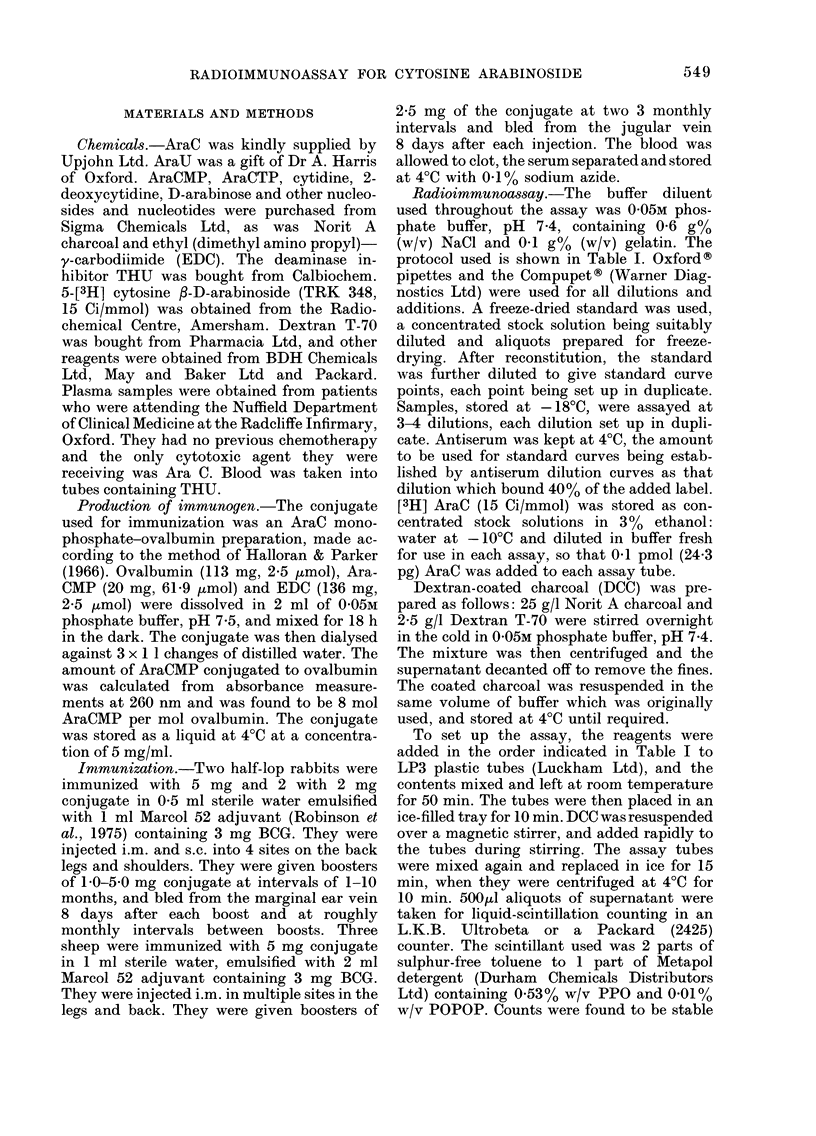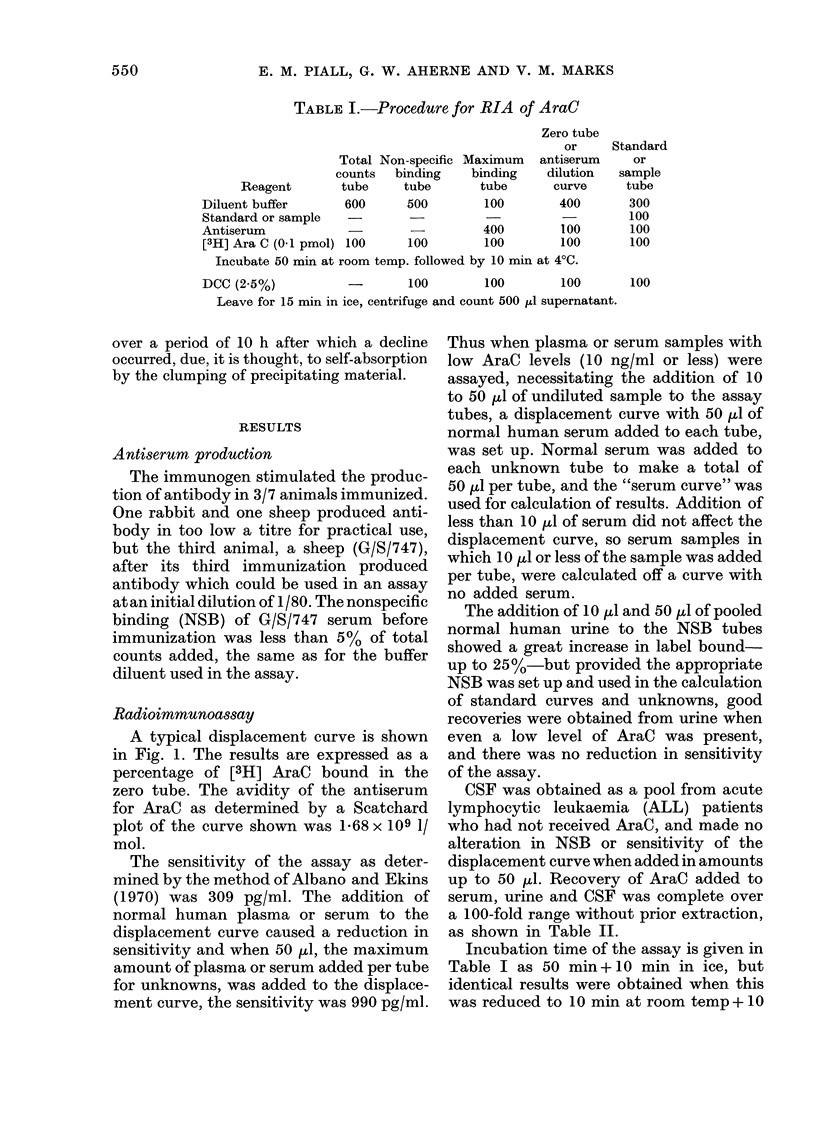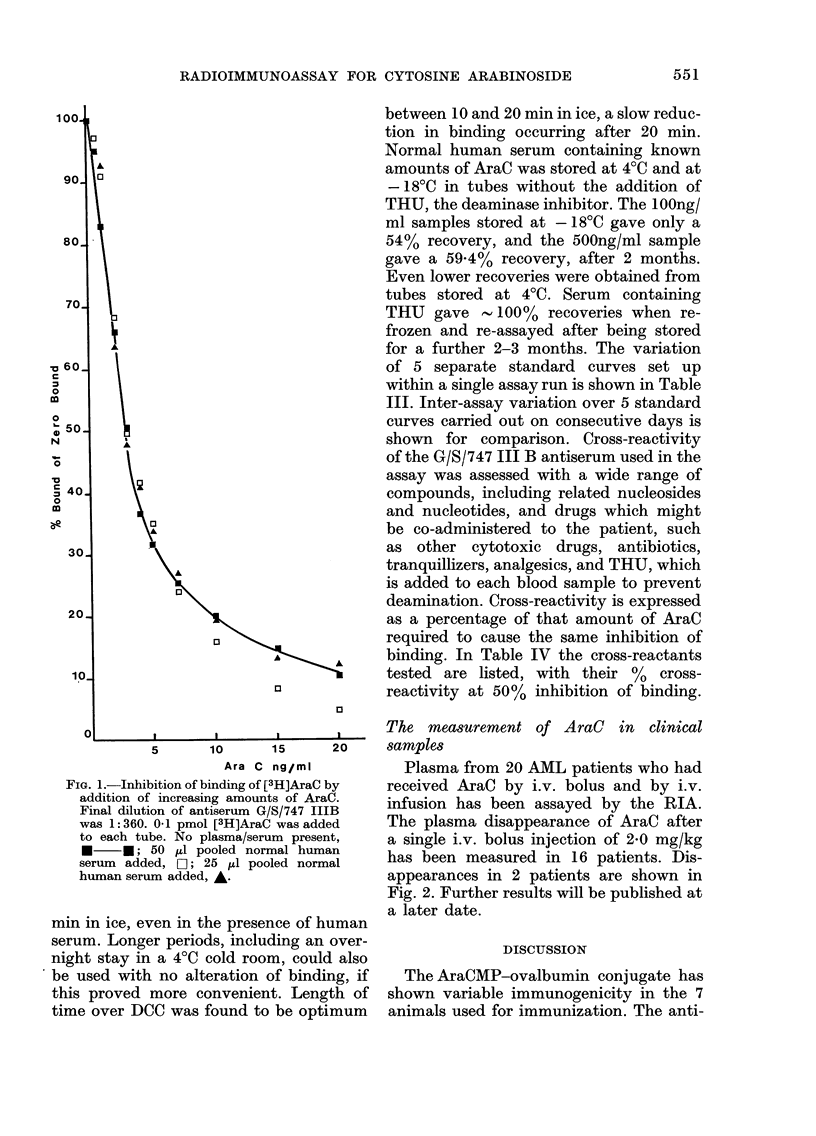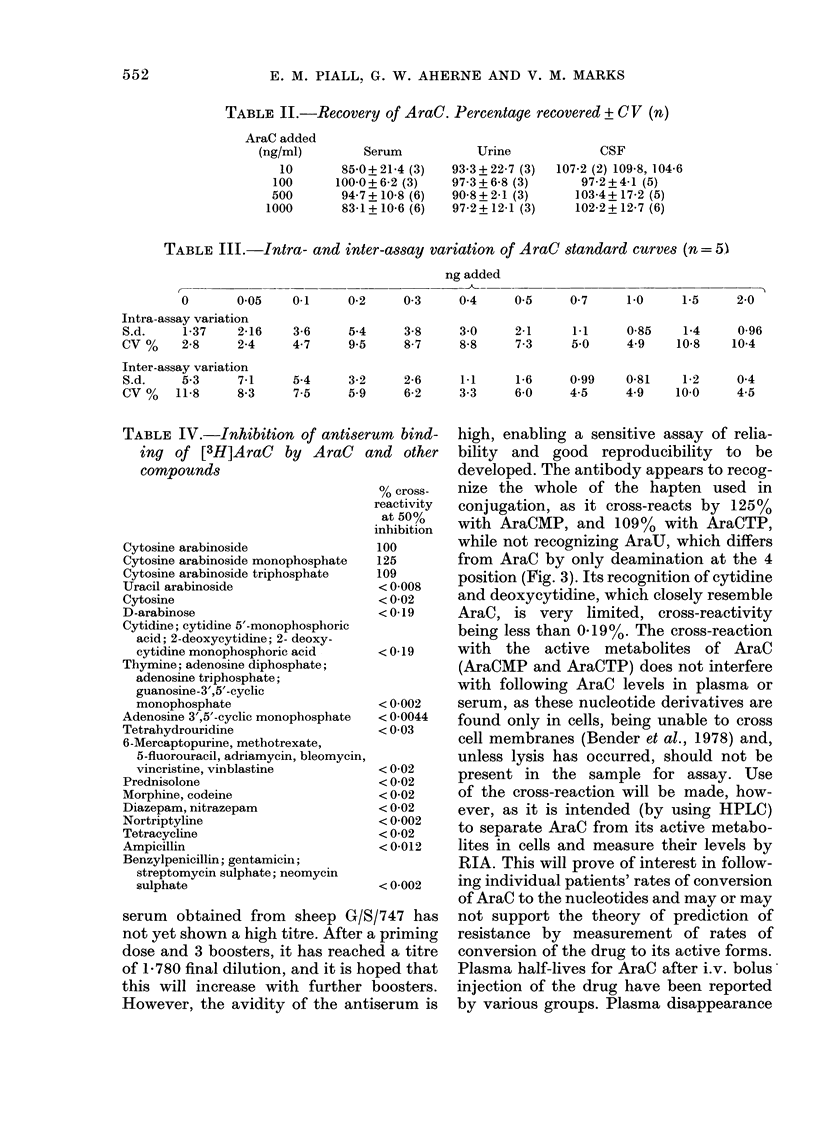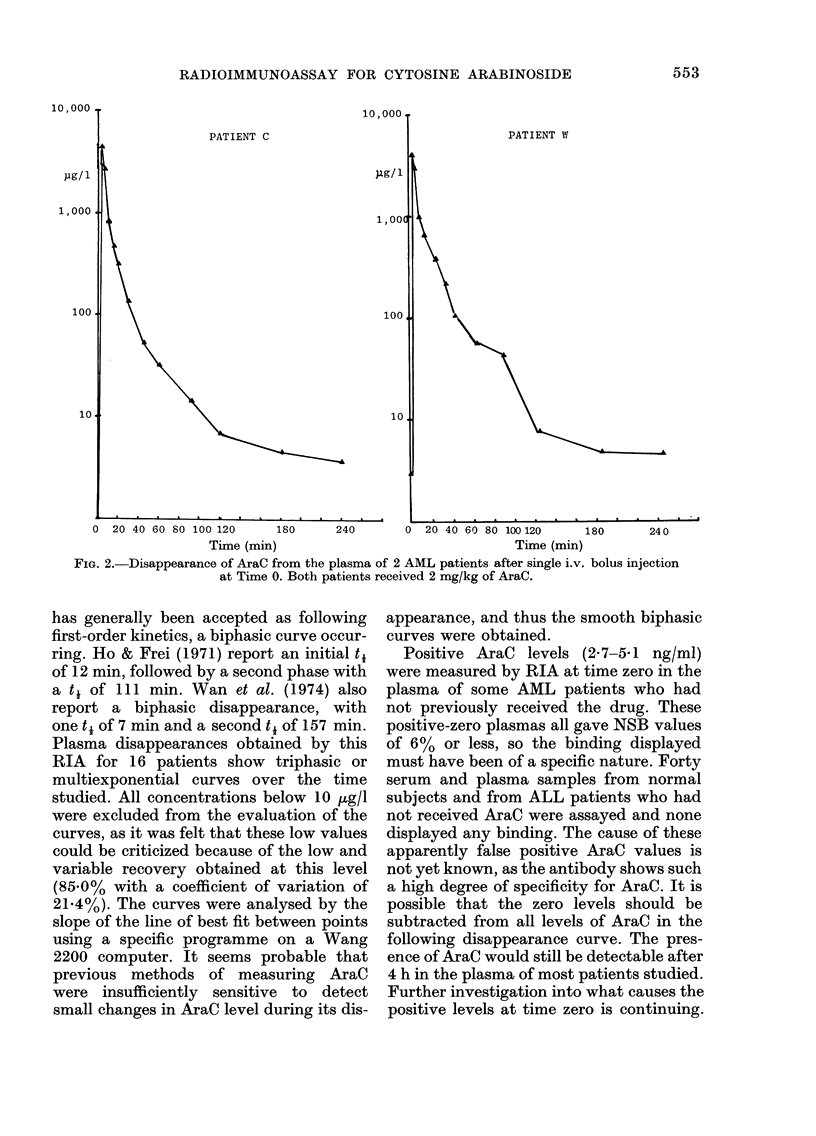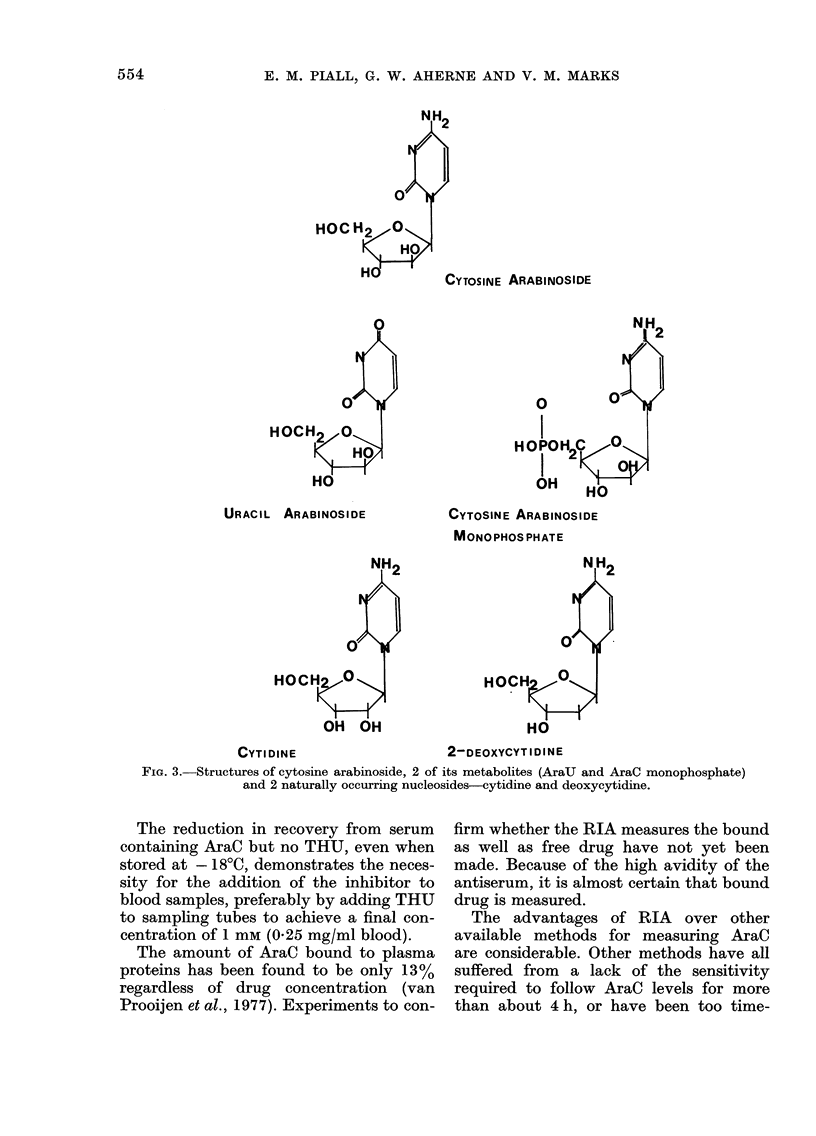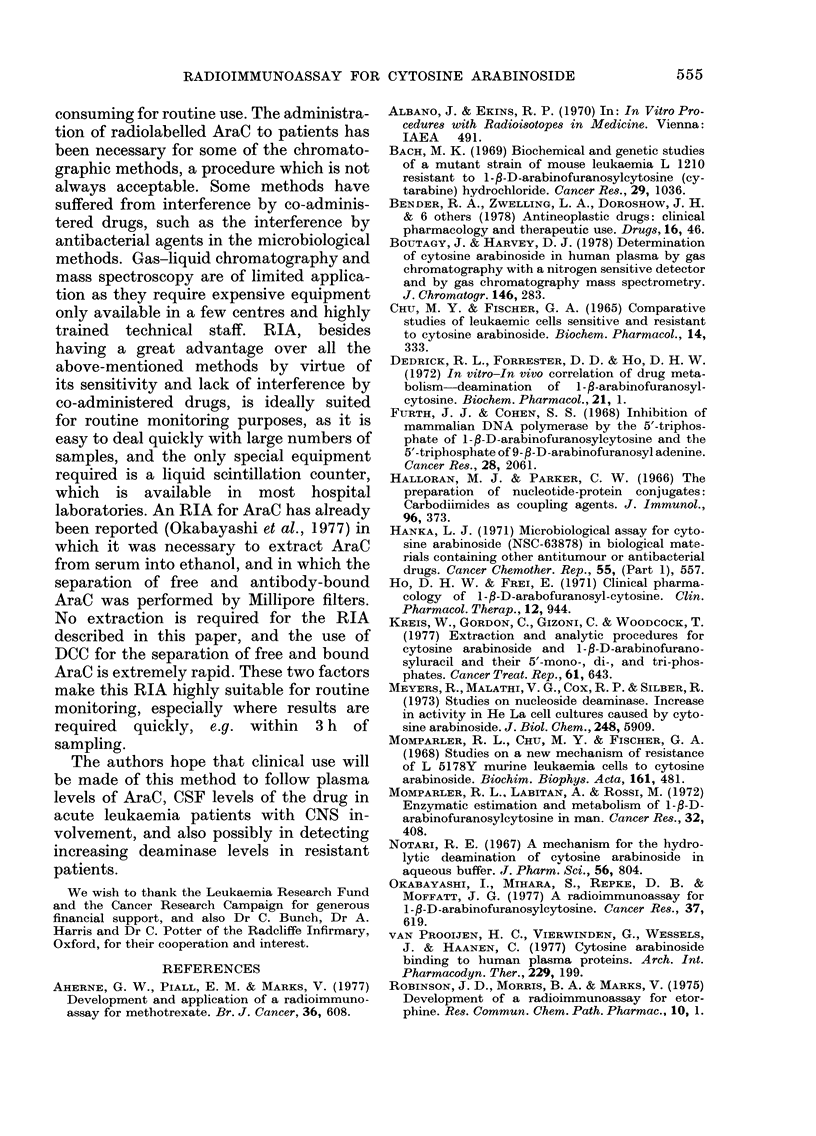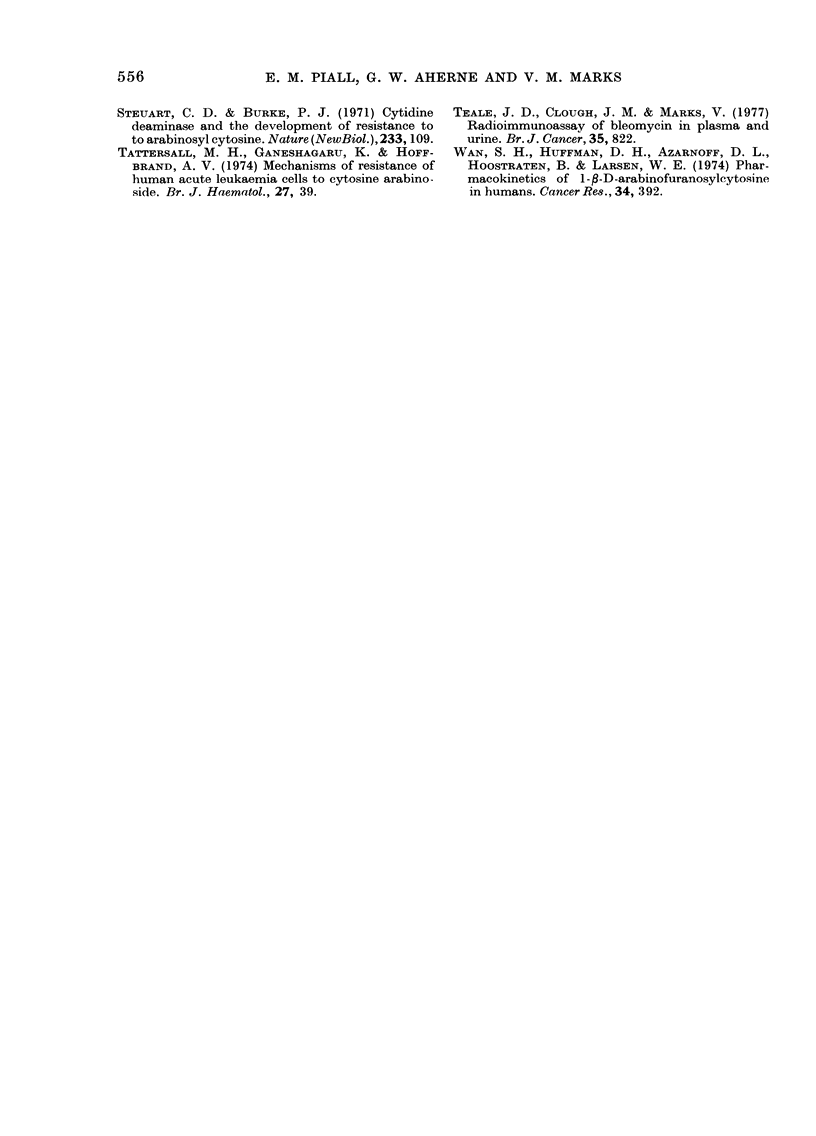# A radioimmunoassay for cytosine arabinoside.

**DOI:** 10.1038/bjc.1979.220

**Published:** 1979-10

**Authors:** E. M. Piall, G. W. Aherne, V. M. Marks

## Abstract

A radioimmunoassay (RIA) for cytosine arabinoside (AraC) has been developed using antiserum raised in a sheep to an AraC monophosphate-ovalbumin conjugate. The antibody shows only 0.008% cross-reactivity with uracil arabinoside (AraU) and low (0.023%) cross-reactivity with other commonly co-administered drugs such as cytotoxic and antibacterial agents, and also a number of naturally occurring nucleosides and nucleotides. It does however cross-react by 125% with AraC monophosphate and by 109% with AraC triphosphate. As little as 1 ng/ml of AraC can be detected in plasma, serum, urine and cerebrospinal fluid (CSF) with no need for prior extraction. This RIA has been used to follow the disappearance of AraC from the plasma of patients receiving the drug.


*Br. J. Cancer* (1979) **40**, 548

### A RADIOIMMUNOASSAY FOR CYTOSINE ARABINOSIDE

E. M. PIALL, G. W. AHERNE AND V. M. MARKS

*From the Division of Clinical Biochemistry, Department of Biochemistry, University of Surrey, Guildford, Surrey*

Received 16 March 1979 Accepted 14 June 1979

**Summary.—A radioimmunoassay (RIA) for cytosine arabinoside (AraC) has been developed using antiserum raised in a sheep to an AraC monophosphate–ovalbumin conjugate. The antibody shows only 0·008% cross-reactivity with uracil arabinoside (AraU) and low (0·023%) cross-reactivity with other commonly co-administered drugs such as cytotoxic and antibacterial agents, and also a number of naturally occurring nucleosides and nucleotides. It does however cross-react by 125% with AraC monophosphate and by 109% with AraC triphosphate. As little as 1 ng/ml of AraC can be detected in plasma, serum, urine and cerebrospinal fluid (CSF) with no need for prior extraction. This RIA has been used to follow the disappearance of AraC from the plasma of patients receiving the drug.**

CYTOSINE ARABINOSIDE (AraC) has been used successfully in the treatment of lymphoma and acute myelogenous leukaemia. The drug is converted rapidly in the blood, either being deaminated to form inactive uracil arabinoside, or phosphorylated by kinases to the active triphosphate form. The pharmacokinetics of AraC have been demonstrated in several studies (Ho & Frei, 1971; Wan *et al.*, 1974; Dedrick *et al.*, 1972). The major cytotoxic effect of AraC triphosphate has been shown to be its competition with deoxycytidine triphosphate for DNA polymerase (Furth & Cohen, 1968). Resistance to AraC has frequently been demonstrated, and several possible causes have been shown to exist. A larger than normal pool size of deoxycytidine nucleotides has been reported in resistant cells (Momparler *et al.*, 1968; Tattersall *et al.*, 1974). A reduced affinity of DNA polymerase for AraCTP has been shown (Bach, 1969) and reduced deoxycytidine kinase levels and increased cytidine deaminase levels have been demonstrated (Chu & Fischer, 1965; Meyers *et al.*, 1973; Steuart & Burke, 1971).

The routine monitoring of circulating AraC during an infusion of the drug or after i.v. bolus injection would seem to be of value in assessing whether therapeutic levels have been reached and whether, after repeated administration, the same dose/blood level is maintained or there is increased deamination.

Such routine monitoring has not been possible with previous methods for measuring AraC. These methods have been microbiological (Hanka, 1971), enzymatic (Momparler *et al.*, 1972), paper or thin-layer chromatography followed by the counting of radiolabelled products or UV absorbance (Ho & Frei, 1971; Dedrick *et al.*, 1972; Notari, 1967) and high-performance liquid chromatography (Kreis *et al.*, 1977). Gas–liquid chromatography and mass spectroscopy have also been used (Boutagy & Harvey, 1978). Immunoassay is probably the best method for routine monitoring for cytotoxic drug levels (Teale *et al.*, 1977; Aherne *et al.*, 1977).

In this paper, the development and application of an RIA for AraC is described which is more sensitive than previous methods for assay of AraC, and is quick to perform.

### MATERIALS AND METHODS

*Chemicals.*—AraC was kindly supplied by Upjohn Ltd. AraU was a gift of Dr A. Harris of Oxford. AraCMP, AraCTP, cytidine, 2-deoxycytidine, D-arabinose and other nucleosides and nucleotides were purchased from Sigma Chemicals Ltd, as was Norit A charcoal and ethyl (dimethyl amino propyl)—$\gamma$-carbodiimide (EDC). The deaminase inhibitor THU was bought from Calbiochem. 5-[$^3$H] cytosine $\beta$-D-arabinoside (TRK 348, 15 Ci/mmol) was obtained from the Radiochemical Centre, Amersham. Dextran T-70 was bought from Pharmacia Ltd, and other reagents were obtained from BDH Chemicals Ltd, May and Baker Ltd and Packard. Plasma samples were obtained from patients who were attending the Nuffield Department of Clinical Medicine at the Radcliffe Infirmary, Oxford. They had no previous chemotherapy and the only cytotoxic agent they were receiving was Ara C. Blood was taken into tubes containing THU.

*Production of immunogen.*—The conjugate used for immunization was an AraC monophosphate–ovalbumin preparation, made according to the method of Halloran & Parker (1966). Ovalbumin (113 mg, 2·5 $\mu$mol), AraCMP (20 mg, 61·9 $\mu$mol) and EDC (136 mg, 2·5 $\mu$mol) were dissolved in 2 ml of 0·05M phosphate buffer, pH 7·5, and mixed for 18 h in the dark. The conjugate was then dialysed against $3 \times 1$ l changes of distilled water. The amount of AraCMP conjugated to ovalbumin was calculated from absorbance measurements at 260 nm and was found to be 8 mol AraCMP per mol ovalbumin. The conjugate was stored as a liquid at 4°C at a concentration of 5 mg/ml.

*Immunization.*—Two half-lop rabbits were immunized with 5 mg and 2 with 2 mg conjugate in 0·5 ml sterile water emulsified with 1 ml Marcol 52 adjuvant (Robinson *et al.*, 1975) containing 3 mg BCG. They were injected i.m. and s.c. into 4 sites on the back legs and shoulders. They were given boosters of 1·0–5·0 mg conjugate at intervals of 1–10 months, and bled from the marginal ear vein 8 days after each boost and at roughly monthly intervals between boosts. Three sheep were immunized with 5 mg conjugate in 1 ml sterile water, emulsified with 2 ml Marcol 52 adjuvant containing 3 mg BCG. They were injected i.m. in multiple sites in the legs and back. They were given boosters of 2·5 mg of the conjugate at two 3 monthly intervals and bled from the jugular vein 8 days after each injection. The blood was allowed to clot, the serum separated and stored at 4°C with 0·1% sodium azide.

*Radioimmunoassay.*—The buffer diluent used throughout the assay was 0·05M phosphate buffer, pH 7·4, containing 0·6 g% (w/v) NaCl and 0·1 g% (w/v) gelatin. The protocol used is shown in Table I. Oxford® pipettes and the Compupet® (Warner Diagnostics Ltd) were used for all dilutions and additions. A freeze-dried standard was used, a concentrated stock solution being suitably diluted and aliquots prepared for freeze-drying. After reconstitution, the standard was further diluted to give standard curve points, each point being set up in duplicate. Samples, stored at −18°C, were assayed at 3–4 dilutions, each dilution set up in duplicate. Antiserum was kept at 4°C, the amount to be used for standard curves being established by antiserum dilution curves as that dilution which bound 40% of the added label. [$^3$H] AraC (15 Ci/mmol) was stored as concentrated stock solutions in 3% ethanol: water at −10°C and diluted in buffer fresh for use in each assay, so that 0·1 pmol (24·3 pg) AraC was added to each assay tube.

Dextran-coated charcoal (DCC) was prepared as follows: 25 g/l Norit A charcoal and 2·5 g/l Dextran T-70 were stirred overnight in the cold in 0·05M phosphate buffer, pH 7·4. The mixture was then centrifuged and the supernatant decanted off to remove the fines. The coated charcoal was resuspended in the same volume of buffer which was originally used, and stored at 4°C until required.

To set up the assay, the reagents were added in the order indicated in Table I to LP3 plastic tubes (Luckham Ltd), and the contents mixed and left at room temperature for 50 min. The tubes were then placed in an ice-filled tray for 10 min. DCC was resuspended over a magnetic stirrer, and added rapidly to the tubes during stirring. The assay tubes were mixed again and replaced in ice for 15 min, when they were centrifuged at 4°C for 10 min. 500$\mu$l aliquots of supernatant were taken for liquid-scintillation counting in an L.K.B. Ultrobeta or a Packard (2425) counter. The scintillant used was 2 parts of sulphur-free toluene to 1 part of Metapol detergent (Durham Chemicals Distributors Ltd) containing 0·53% w/v PPO and 0·01% w/v POPOP. Counts were found to be stable

TABLE I.—*Procedure for RIA of AraC*

| Reagent | Total counts tube | Non-specific binding tube | Maximum binding tube | Zero tube or antiserum dilution curve | Standard or sample tube |
|---|---|---|---|---|---|
| Diluent buffer | 600 | 500 | 100 | 400 | 300 |
| Standard or sample | — | — | — | — | 100 |
| Antiserum | — | — | 400 | 100 | 100 |
| [$^3$H] Ara C (0·1 pmol) | 100 | 100 | 100 | 100 | 100 |
| Incubate 50 min at room temp. followed by 10 min at 4°C. | | | | | |
| DCC (2·5%) | — | 100 | 100 | 100 | 100 |
| Leave for 15 min in ice, centrifuge and count 500 $\mu$l supernatant. | | | | | |

over a period of 10 h after which a decline occurred, due, it is thought, to self-absorption by the clumping of precipitating material.

### RESULTS

### *Antiserum production*

The immunogen stimulated the production of antibody in 3/7 animals immunized. One rabbit and one sheep produced antibody in too low a titre for practical use, but the third animal, a sheep (G/S/747), after its third immunization produced antibody which could be used in an assay at an initial dilution of 1/80. The nonspecific binding (NSB) of G/S/747 serum before immunization was less than 5% of total counts added, the same as for the buffer diluent used in the assay.

### *Radioimmunoassay*

A typical displacement curve is shown in Fig. 1. The results are expressed as a percentage of [$^3$H] AraC bound in the zero tube. The avidity of the antiserum for AraC as determined by a Scatchard plot of the curve shown was $1·68 \times 10^9$ l/mol.

The sensitivity of the assay as determined by the method of Albano and Ekins (1970) was 309 pg/ml. The addition of normal human plasma or serum to the displacement curve caused a reduction in sensitivity and when 50 $\mu$l, the maximum amount of plasma or serum added per tube for unknowns, was added to the displacement curve, the sensitivity was 990 pg/ml. Thus when plasma or serum samples with low AraC levels (10 ng/ml or less) were assayed, necessitating the addition of 10 to 50 $\mu$l of undiluted sample to the assay tubes, a displacement curve with 50 $\mu$l of normal human serum added to each tube, was set up. Normal serum was added to each unknown tube to make a total of 50 $\mu$l per tube, and the "serum curve" was used for calculation of results. Addition of less than 10 $\mu$l of serum did not affect the displacement curve, so serum samples in which 10 $\mu$l or less of the sample was added per tube, were calculated off a curve with no added serum.

The addition of 10 $\mu$l and 50 $\mu$l of pooled normal human urine to the NSB tubes showed a great increase in label bound—up to 25%—but provided the appropriate NSB was set up and used in the calculation of standard curves and unknowns, good recoveries were obtained from urine when even a low level of AraC was present, and there was no reduction in sensitivity of the assay.

CSF was obtained as a pool from acute lymphocytic leukaemia (ALL) patients who had not received AraC, and made no alteration in NSB or sensitivity of the displacement curve when added in amounts up to 50 $\mu$l. Recovery of AraC added to serum, urine and CSF was complete over a 100-fold range without prior extraction, as shown in Table II.

Incubation time of the assay is given in Table I as 50 min + 10 min in ice, but identical results were obtained when this was reduced to 10 min at room temp + 10

FIG. 1.—Inhibition of binding of [$^{3}$H]AraC by addition of increasing amounts of AraC. Final dilution of antiserum G/S/747 IIIB was 1:360. 0·1 pmol [$^{3}$H]AraC was added to each tube. No plasma/serum present, ■——■; 50 $\mu$l pooled normal human serum added, □; 25 $\mu$l pooled normal human serum added, ▲.

min in ice, even in the presence of human serum. Longer periods, including an overnight stay in a 4°C cold room, could also be used with no alteration of binding, if this proved more convenient. Length of time over DCC was found to be optimum between 10 and 20 min in ice, a slow reduction in binding occurring after 20 min. Normal human serum containing known amounts of AraC was stored at 4°C and at −18°C in tubes without the addition of THU, the deaminase inhibitor. The 100ng/ml samples stored at −18°C gave only a 54% recovery, and the 500ng/ml sample gave a 59·4% recovery, after 2 months. Even lower recoveries were obtained from tubes stored at 4°C. Serum containing THU gave ~100% recoveries when re-frozen and re-assayed after being stored for a further 2–3 months. The variation of 5 separate standard curves set up within a single assay run is shown in Table III. Inter-assay variation over 5 standard curves carried out on consecutive days is shown for comparison. Cross-reactivity of the G/S/747 III B antiserum used in the assay was assessed with a wide range of compounds, including related nucleosides and nucleotides, and drugs which might be co-administered to the patient, such as other cytotoxic drugs, antibiotics, tranquillizers, analgesics, and THU, which is added to each blood sample to prevent deamination. Cross-reactivity is expressed as a percentage of that amount of AraC required to cause the same inhibition of binding. In Table IV the cross-reactants tested are listed, with their % cross-reactivity at 50% inhibition of binding.

*The measurement of AraC in clinical samples*

Plasma from 20 AML patients who had received AraC by i.v. bolus and by i.v. infusion has been assayed by the RIA. The plasma disappearance of AraC after a single i.v. bolus injection of 2·0 mg/kg has been measured in 16 patients. Disappearances in 2 patients are shown in Fig. 2. Further results will be published at a later date.

DISCUSSION

The AraCMP–ovalbumin conjugate has shown variable immunogenicity in the 7 animals used for immunization. The anti-

TABLE II.—*Recovery of AraC. Percentage recovered* ± *CV* (*n*)

| AraC added (ng/ml) | Serum | Urine | CSF |
|---|---|---|---|
| 10 | 85·0 ± 21·4 (3) | 93·3 ± 22·7 (3) | 107·2 (2) 109·8, 104·6 |
| 100 | 100·0 ± 6·2 (3) | 97·3 ± 6·8 (3) | 97·2 ± 4·1 (5) |
| 500 | 94·7 ± 10·8 (6) | 90·8 ± 2·1 (3) | 103·4 ± 17·2 (5) |
| 1000 | 83·1 ± 10·6 (6) | 97·2 ± 12·1 (3) | 102·2 ± 12·7 (6) |

TABLE III.—*Intra- and inter-assay variation of AraC standard curves* ($n = 5$)

| | ng added | | | | | | | | | | |
|---|---|---|---|---|---|---|---|---|---|---|---|
| | 0 | 0·05 | 0·1 | 0·2 | 0·3 | 0·4 | 0·5 | 0·7 | 1·0 | 1·5 | 2·0 |
| Intra-assay variation | | | | | | | | | | | |
| S.d. | 1·37 | 2·16 | 3·6 | 5·4 | 3·8 | 3·0 | 2·1 | 1·1 | 0·85 | 1·4 | 0·96 |
| CV % | 2·8 | 2·4 | 4·7 | 9·5 | 8·7 | 8·8 | 7·3 | 5·0 | 4·9 | 10·8 | 10·4 |
| Inter-assay variation | | | | | | | | | | | |
| S.d. | 5·3 | 7·1 | 5·4 | 3·2 | 2·6 | 1·1 | 1·6 | 0·99 | 0·81 | 1·2 | 0·4 |
| CV % | 11·8 | 8·3 | 7·5 | 5·9 | 6·2 | 3·3 | 6·0 | 4·5 | 4·9 | 10·0 | 4·5 |

TABLE IV.—*Inhibition of antiserum binding of* [$^3$*H*]*AraC by AraC and other compounds*

| | % cross-reactivity at 50% inhibition |
|---|---|
| Cytosine arabinoside | 100 |
| Cytosine arabinoside monophosphate | 125 |
| Cytosine arabinoside triphosphate | 109 |
| Uracil arabinoside | < 0·008 |
| Cytosine | < 0·02 |
| D-arabinose | < 0·19 |
| Cytidine; cytidine 5′-monophosphoric acid; 2-deoxycytidine; 2- deoxy-cytidine monophosphoric acid | < 0·19 |
| Thymine; adenosine diphosphate; adenosine triphosphate; guanosine-3′,5′-cyclic monophosphate | < 0·002 |
| Adenosine 3′,5′-cyclic monophosphate | < 0·0044 |
| Tetrahydrouridine | < 0·03 |
| 6-Mercaptopurine, methotrexate, 5-fluorouracil, adriamycin, bleomycin, vincristine, vinblastine | < 0·02 |
| Prednisolone | < 0·02 |
| Morphine, codeine | < 0·02 |
| Diazepam, nitrazepam | < 0·02 |
| Nortriptyline | < 0·002 |
| Tetracycline | < 0·02 |
| Ampicillin | < 0·012 |
| Benzylpenicillin; gentamicin; streptomycin sulphate; neomycin sulphate | < 0·002 |

serum obtained from sheep G/S/747 has not yet shown a high titre. After a priming dose and 3 boosters, it has reached a titre of 1·780 final dilution, and it is hoped that this will increase with further boosters. However, the avidity of the antiserum is high, enabling a sensitive assay of reliability and good reproducibility to be developed. The antibody appears to recognize the whole of the hapten used in conjugation, as it cross-reacts by 125% with AraCMP, and 109% with AraCTP, while not recognizing AraU, which differs from AraC by only deamination at the 4 position (Fig. 3). Its recognition of cytidine and deoxycytidine, which closely resemble AraC, is very limited, cross-reactivity being less than 0·19%. The cross-reaction with the active metabolites of AraC (AraCMP and AraCTP) does not interfere with following AraC levels in plasma or serum, as these nucleotide derivatives are found only in cells, being unable to cross cell membranes (Bender *et al.*, 1978) and, unless lysis has occurred, should not be present in the sample for assay. Use of the cross-reaction will be made, however, as it is intended (by using HPLC) to separate AraC from its active metabolites in cells and measure their levels by RIA. This will prove of interest in following individual patients' rates of conversion of AraC to the nucleotides and may or may not support the theory of prediction of resistance by measurement of rates of conversion of the drug to its active forms. Plasma half-lives for AraC after i.v. bolus injection of the drug have been reported by various groups. Plasma disappearance

FIG. 2.—Disappearance of AraC from the plasma of 2 AML patients after single i.v. bolus injection at Time 0. Both patients received 2 mg/kg of AraC.

has generally been accepted as following first-order kinetics, a biphasic curve occurring. Ho & Frei (1971) report an initial $t_{\frac{1}{2}}$ of 12 min, followed by a second phase with a $t_{\frac{1}{2}}$ of 111 min. Wan *et al.* (1974) also report a biphasic disappearance, with one $t_{\frac{1}{2}}$ of 7 min and a second $t_{\frac{1}{2}}$ of 157 min. Plasma disappearances obtained by this RIA for 16 patients show triphasic or multiexponential curves over the time studied. All concentrations below 10 $\mu$g/l were excluded from the evaluation of the curves, as it was felt that these low values could be criticized because of the low and variable recovery obtained at this level (85·0% with a coefficient of variation of 21·4%). The curves were analysed by the slope of the line of best fit between points using a specific programme on a Wang 2200 computer. It seems probable that previous methods of measuring AraC were insufficiently sensitive to detect small changes in AraC level during its disappearance, and thus the smooth biphasic curves were obtained.

Positive AraC levels (2·7–5·1 ng/ml) were measured by RIA at time zero in the plasma of some AML patients who had not previously received the drug. These positive-zero plasmas all gave NSB values of 6% or less, so the binding displayed must have been of a specific nature. Forty serum and plasma samples from normal subjects and from ALL patients who had not received AraC were assayed and none displayed any binding. The cause of these apparently false positive AraC values is not yet known, as the antibody shows such a high degree of specificity for AraC. It is possible that the zero levels should be subtracted from all levels of AraC in the following disappearance curve. The presence of AraC would still be detectable after 4 h in the plasma of most patients studied. Further investigation into what causes the positive levels at time zero is continuing.

FIG. 3.—Structures of cytosine arabinoside, 2 of its metabolites (AraU and AraC monophosphate) and 2 naturally occurring nucleosides—cytidine and deoxycytidine.

The reduction in recovery from serum containing AraC but no THU, even when stored at −18°C, demonstrates the necessity for the addition of the inhibitor to blood samples, preferably by adding THU to sampling tubes to achieve a final concentration of 1 mM (0·25 mg/ml blood).

The amount of AraC bound to plasma proteins has been found to be only 13% regardless of drug concentration (van Prooijen *et al.*, 1977). Experiments to confirm whether the RIA measures the bound as well as free drug have not yet been made. Because of the high avidity of the antiserum, it is almost certain that bound drug is measured.

The advantages of RIA over other available methods for measuring AraC are considerable. Other methods have all suffered from a lack of the sensitivity required to follow AraC levels for more than about 4 h, or have been too timeconsuming for routine use. The administration of radiolabelled AraC to patients has been necessary for some of the chromatographic methods, a procedure which is not always acceptable. Some methods have suffered from interference by co-administered drugs, such as the interference by antibacterial agents in the microbiological methods. Gas–liquid chromatography and mass spectroscopy are of limited application as they require expensive equipment only available in a few centres and highly trained technical staff. RIA, besides having a great advantage over all the above-mentioned methods by virtue of its sensitivity and lack of interference by co-administered drugs, is ideally suited for routine monitoring purposes, as it is easy to deal quickly with large numbers of samples, and the only special equipment required is a liquid scintillation counter, which is available in most hospital laboratories. An RIA for AraC has already been reported (Okabayashi *et al.*, 1977) in which it was necessary to extract AraC from serum into ethanol, and in which the separation of free and antibody-bound AraC was performed by Millipore filters. No extraction is required for the RIA described in this paper, and the use of DCC for the separation of free and bound AraC is extremely rapid. These two factors make this RIA highly suitable for routine monitoring, especially where results are required quickly, *e.g.* within 3 h of sampling.

The authors hope that clinical use will be made of this method to follow plasma levels of AraC, CSF levels of the drug in acute leukaemia patients with CNS involvement, and also possibly in detecting increasing deaminase levels in resistant patients.

We wish to thank the Leukaemia Research Fund and the Cancer Research Campaign for generous financial support, and also Dr C. Bunch, Dr A. Harris and Dr C. Potter of the Radcliffe Infirmary, Oxford, for their cooperation and interest.

### REFERENCES

Aherne, G. W., Piall, E. M. & Marks, V. (1977) Development and application of a radioimmunoassay for methotrexate. *Br. J. Cancer*, **36**, 608.

Albano, J. & Ekins, R. P. (1970) In: *In Vitro Procedures with Radioisotopes in Medicine*. Vienna: IAEA 491.

Bach, M. K. (1969) Biochemical and genetic studies of a mutant strain of mouse leukaemia L 1210 resistant to 1-β-D-arabinofuranosylcytosine (cytarabine) hydrochloride. *Cancer Res.*, **29**, 1036.

Bender, R. A., Zwelling, L. A., Doroshow, J. H. & 6 others (1978) Antineoplastic drugs: clinical pharmacology and therapeutic use. *Drugs*, **16**, 46.

Boutagy, J. & Harvey, D. J. (1978) Determination of cytosine arabinoside in human plasma by gas chromatography with a nitrogen sensitive detector and by gas chromatography mass spectrometry. *J. Chromatogr.* **146**, 283.

Chu, M. Y. & Fischer, G. A. (1965) Comparative studies of leukaemic cells sensitive and resistant to cytosine arabinoside. *Biochem. Pharmacol.*, **14**, 333.

Dedrick, R. L., Forrester, D. D. & Ho, D. H. W. (1972) *In vitro–In vivo* correlation of drug metabolism—deamination of 1-β-arabinofuranosylcytosine. *Biochem. Pharmacol.*, **21**, 1.

Furth, J. J. & Cohen, S. S. (1968) Inhibition of mammalian DNA polymerase by the 5′-triphosphate of 1-β-D-arabinofuranosylcytosine and the 5′-triphosphate of 9-β-D-arabinofuranosyl adenine. *Cancer Res.*, **28**, 2061.

Halloran, M. J. & Parker, C. W. (1966) The preparation of nucleotide-protein conjugates: Carbodiimides as coupling agents. *J. Immunol.*, **96**, 373.

Hanka, L. J. (1971) Microbiological assay for cytosine arabinoside (NSC-63878) in biological materials containing other antitumour or antibacterial drugs. *Cancer Chemother. Rep.*, **55**, (Part 1), 557.

Ho, D. H. W. & Frei, E. (1971) Clinical pharmacology of 1-β-D-arabofuranosyl-cytosine. *Clin. Pharmacol. Therap.*, **12**, 944.

Kreis, W., Gordon, C., Gizoni, C. & Woodcock, T. (1977) Extraction and analytic procedures for cytosine arabinoside and 1-β-D-arabinofuranosyluracil and their 5′-mono-, di-, and tri-phosphates. *Cancer Treat. Rep.*, **61**, 643.

Meyers, R., Malathi, V. G., Cox, R. P. & Silber, R. (1973) Studies on nucleoside deaminase. Increase in activity in He La cell cultures caused by cytosine arabinoside. *J. Biol. Chem.*, **248**, 5909.

Momparler, R. L., Chu, M. Y. & Fischer, G. A. (1968) Studies on a new mechanism of resistance of L 5178Y murine leukaemia cells to cytosine arabinoside. *Biochim. Biophys. Acta*, **161**, 481.

Momparler, R. L., Labitan, A. & Rossi, M. (1972) Enzymatic estimation and metabolism of 1-β-D-arabinofuranosylcytosine in man. *Cancer Res.*, **32**, 408.

Notari, R. E. (1967) A mechanism for the hydrolytic deamination of cytosine arabinoside in aqueous buffer. *J. Pharm. Sci.*, **56**, 804.

Okabayashi, I., Mihara, S., Repke, D. B. & Moffatt, J. G. (1977) A radioimmunoassay for 1-β-D-arabinofuranosylcytosine. *Cancer Res.*, **37**, 619.

van Prooijen, H. C., Vierwinden, G., Wessels, J. & Haanen, C. (1977) Cytosine arabinoside binding to human plasma proteins. *Arch. Int. Pharmacodyn. Ther.*, **229**, 199.

Robinson, J. D., Morris, B. A. & Marks, V. (1975) Development of a radioimmunoassay for etorphine. *Res. Commun. Chem. Path. Pharmac.*, **10**, 1.

STEUART, C. D. & BURKE, P. J. (1971) Cytidine deaminase and the development of resistance to to arabinosyl cytosine. *Nature (New Biol.)*, **233**, 109.
TATTERSALL, M. H., GANESHAGARU, K. & HOFFBRAND, A. V. (1974) Mechanisms of resistance of human acute leukaemia cells to cytosine arabinoside. *Br. J. Haematol.*, **27**, 39.
TEALE, J. D., CLOUGH, J. M. & MARKS, V. (1977) Radioimmunoassay of bleomycin in plasma and urine. *Br. J. Cancer*, **35**, 822.
WAN, S. H., HUFFMAN, D. H., AZARNOFF, D. L., HOOSTRATEN, B. & LARSEN, W. E. (1974) Pharmacokinetics of 1-β-D-arabinofuranosylcytosine in humans. *Cancer Res.*, **34**, 392.